# Role of gait speed and grip strength in predicting 10-year cognitive decline among community-dwelling older people

**DOI:** 10.1186/s12877-019-1199-7

**Published:** 2019-07-05

**Authors:** Ming-Yueh Chou, Yukiko Nishita, Takeshi Nakagawa, Chikako Tange, Makiko Tomida, Hiroshi Shimokata, Rei Otsuka, Liang-Kung Chen, Hidenori Arai

**Affiliations:** 1 Center for Geriatrics and Gerontology, Kaohsiung Veterans General Hospital, No.386, Dazhong 1st Rd., Zuoying Dist., Kaohsiung City, 813 Taiwan, Republic of China; 2 Aging and Health Research Center, National Yang Ming University, No.155, Sec.2, Linong Street, Taipei, 112 Taiwan, Republic of China; 3 Department of Geriatric Medicine, National Yang Ming University School of Medicine, No.155, Sec.2, Linong Street, Taipei, 112 Taiwan, Republic of China; 4 National Center for Geriatrics and Gerontology, 7-430 Morioka, Obu, Aichi 474-8511 Japan; 5 Chia Nan University of Pharmacy and Science, No.60, Sec. 1, Erren Rd., Rende Dist., Tainan City, 717 Taiwan, Republic of China; 6 Department of Epidemiology of Aging, National Center for Geriatrics and Gerontology, 7-430 Morioka, Obu, Aichi 474-8511 Japan; 7 Section of NILS-LSA, Center for Gerontology and Social Science, National Center for Geriatrics and Gerontology, 7-430, Morioka-cho, Obu, Aichi 474-8511 Japan; 8 The Japan Society for the Promotion of Science, 5-3-1 Kojimachi, Chiyoda-ku, Tokyo, Japan; 9 Graduate School of Nutritional Sciences, Nagoya University of Arts and Sciences, Furo-cho, Chikusa-ku, Nagoya, 464-8601 Japan; 10 Center for Geriatrics and Gerontology, Taipei Veterans General Hospital, No.201, Sec. 2, Shipai Rd., Beitou District, Taipei City, 112 Taiwan, Republic of China

**Keywords:** Gait speed, Handgrip strength, Cognition, MMSE, DSST

## Abstract

**Background:**

The gait speed and handgrip strength represented the core determinants of physical frailty and sarcopenia, which were reported to be associated with cognitive impairment and decline. Different physical measures might differentially affect cognitive changes, such as higher-level cognitive change and global cognitive decline. This study examined the differential associations of gait speed and handgrip strength with 10-year cognitive changes among community-dwelling older people.

**Methods:**

Participants aged 60 years and over living in the community were invited for study. Gait speed and handgrip strength were classified into 5 groups based on quintiles at baseline. Cognitive functions were assessed using the Mini-Mental State Examination (MMSE) and Digit Symbol Substitution Test (DSST) every 2 years from baseline for a period of 10 years. Linear mixed effects models were used to determine the role of gait speed and handgrip strength in the prediction of 10-year cognitive changes by adjusting covariates, including age, gender, education, depressive symptoms, marital status, smoking status, instrumental activities of daily life (IADL), Charlson Comorbidity Index (CCI), and body mass index (BMI) at baseline.

**Results:**

A total of 1096 participants were enrolled in the study. The mean age was 69.4 ± 5.8 years and 50.9% were male. The slowest gait speed group showed a significantly greater decline in the DSST scores over 10 years than the highest group (estimate = 0.28 and *P* = 0.003), but not in the MMSE scores (estimate = 0.05 and *P* = 0.078). The lowest handgrip strength group showed a significantly greater decline in the MMSE scores than the highest group (estimate = 0.06 and *P* = 0.039) and in the DSST scores than the highest two quintiles (estimate = 0.20 and *P* = 0.033 for the fourth quintile; estimate = 0.20 and *P* = 0.040 for the highest quintile) over 10-year follow-up.

**Conclusions:**

A slow gait speed could predict 10-year cognitive decline using DSST, and a low handgrip strength could predict 10-year cognitive decline using MMSE in addition to DSST. Thus both physical measures are lined to cognitive decline but there may be different mechanisms between brain and physical functions.

## Background

Dementia is a global health issue and is accompanied by a loss of cognitive capacity that is severe enough to affect social participation and the capacity for independent living [1, 2]. Several studies have reported that cognitive decline may predict the development of dementia [3, 4]. Identifying the early markers of cognitive decline may help identify persons with high risks of dementia and allow for the prevention or delay of the process of developing dementia [5, 6]. Potentially modifiable risk factors in the development of cognitive decline or impairment have been well established, and among these, physical activity has been considered a factor of interest in recent studies [7–9]. Additionally, several studies have revealed that physical function precedes cognitive decline [10–13].

The gait speed and handgrip strength represented the core determinants of physical frailty and sarcopenia, which were reported to be associated with cognitive impairment and decline [12, 14, 15]. Previous studies have also shown that physical function parameters, such as gait speed and handgrip strength, are associated with cognitive function in older people [15–17]. Therefore, we explored gait speed and handgrip strength as physical function indicators in this study.

Recent review articles have shown that most studies explore cognitive decline by using the Mini-Mental State Examination (MMSE) [10, 11], which is widely used as a tool to screen for global cognitive impairment. However, the MMSE assesses the basic level of cognitive functions and may be insensitive to changes in well-functioning community-dwelling adults. Therefore, we also used the Digit Symbol Substitution Test (DSST) as a more sensitive indicator of cognitive function. The DSST measures general and unspecific processing speed and is more sensitive to slight changes in higher-level cognition than the MMSE [18]. The DSST refers to how quickly one can accurately process new inputs from outside and retrieve stored information from memory [19]. Processing speed, by playing a central role in cognition, is affected by the changes experienced chronologically and can predict incident dementia [20]. Moreover, recent studies have suggested that interventions could improve cognitive functioning by increasing information processing speed among older people [21, 22].

Clouston et al., reviewed that gait speed is associated with a change of fluid cognition while handgrip strength is correlated with changes in global cognitive function [23]. Different physical measures might differentially affect cognitive changes, such as higher-level cognitive change and global cognitive decline. Therefore, the main aim of this study was to evaluate the different effects of gait speed and handgrip strength on 10-year cognitive changes by using the DSST and MMSE among community-dwelling older people.

## Methods

### Study design

The data for the present study was obtained as part of the National Institute for Longevity Science – Longitudinal Study of Aging (NILS-LSA) [24]. The NILS-LSA was a population-based prospective cohort study: community-dwelling adults living in the neighborhood of the institute (Obu City and Higashiura Town) and who were 40–79 years old at baseline were invited into the study based on a random sample stratified by age and gender between November 1997 and April 2000. The baseline sample included 2267 participants, and a follow up was conducted every 2 years. When participants could not attend the follow up investigation, new age-gender matched subjects aged up to 79 years old were randomly selected.

In the present study, due to a lack of data from the MMSE in the first wave, we selected a sample of participants in the second wave (April 2000 to May 2002; *N* = 2259). Participants with the following conditions were excluded: (1) younger than 60 years old (*n* = 1114) and (2) cognitive impairment (MMSE scores less than 24) (*n* = 37) or no cognitive information (*n* = 12). In total, 1096 participants were enrolled in the study [mean age 69.4 ± standard deviation (SD) 5.8 years, 50.9% male]. In addition, participants without assessment of gait speed or handgrip strength were excluded for each analysis. The study protocol was approved by the Committee of Ethics of Human Research of the National Center for Geriatrics and Gerontology. Written informed consent was obtained from all participants. All methods were performed in accordance with relevant guidelines and regulations.

### Assessment of physical function (using the 2^nd^ wave as baseline)

In this study, the baseline measurements of physical function included gait speed and handgrip strength. The current cut-off points of gait speed and handgrip strength as the determinants of frailty and sarcopenia were suggested by the lowest quintile or quartile according to the study population, and it is also practical to implement the parameters according to the cut-off point for clinical practice rather than continuous variables [25, 26]. Therefore, we used the quintiles of physical function from the study population. Gait speed was assessed by the walking analysis system (YW-3, Yagami Co., Aichi, Japan) [27]. Participants were asked to walk at a comfortable speed on an 11-m straight walkway, including 1 m for acceleration and deceleration. Light sensors were used for the start and end points to record the time taken to walk 10 m, and habitual gait speed was measured by calculating this in meters per second. Due to the significant difference of gait speeds between genders, all participants were classified into quintiles according to gender (Q1 as the lowest quintile and Q5 as the highest quintile: males ≦1.12; 1.13–1.25; 1.26–1.37; 1.38–1.47; and > 1.48, m/s; and females ≦1.13; 1.14–1.22; 1.23–1.32; 1.33–1.42; and > 1.43, m/s). Handgrip strength was measured using a handgrip dynamometer (Takei Co., Niigata, Japan) calibrated in kg [28]. The participants stood and extended their elbows to hold a handgrip dynamometer. Two trials of both hands were tested alternately, and the maximal value was used as the measurement result. Then, all participants were classified into quintiles according to gender (Q1 as the lowest quintile and Q5 as the highest quintile: males≦30.70; 30.71–33.90; 33.91–37.40; 37.41–41.25; and > 41.25 kg; females ≦18.50; 18.51–20.90; 20.91–23.10; 23.11–25.70; and > 25.70 kg).

### Assessment of cognitive function (from 2^nd^ to 7^th^ waves)

Cognitive function was assessed from the second to seventh waves using the Japanese version of the MMSE (scores 0–30, higher score meaning better cognitive function) and the DSST (scores 0–93; higher scores mean better cognitive function) in interviews with a psychologist or psychology graduate students [29, 30]. The DSST was calculated from the Japanese Wechsler Adult Intelligence Scale–Revised Short Form (JWAIS-R-SF), which is a popular tool for assessing intelligence [31]. The DSST measured the information processing speed. Participants were asked to write down the symbol that corresponded to a given number, as many as they could in 90 s.

Given that the number of participants who scored below the cut-off score of 23/24 on the MMSE were small across waves (*n* = 17—31), we used scores of the MMSE as well as the DSST as continuous variables.

### Covariates (2^nd^ wave as baseline)

Using the 2^nd^ wave as baseline, some covariates, including age, gender (0 = male; 1 = female), education level (years), marital status (0 = not married; 1 = married), smoking status (0 = nonsmoker; 1 = smoker),and depressive symptoms (using Center for Epidemiologic Studies Depression Scale (CESD), 0 = non-depressive symptoms; 1 = depressive symptoms, defined as 16 or greater) [32], were collected by a self-administered questionnaire. Body mass index (BMI) was calculated as weight in kilograms divided by the square of height in meters. Instrumental activities of daily living (IADLs) were assessed using the Instrumental Self-Maintenance subscale of the Tokyo Metropolitan Institute of Gerontology Index of Competence (TMIG-IC) [33], and comorbidity was presented as the Charlson Comorbidity Index (CCI) [34].

### Statistical analysis

In this study, all continuous variables are presented as the mean ± standard deviation (SD), and categorical data are presented as numbers (percentages). Student’s t-test or one-way ANOVA was used for the comparison of continuous variables among groups, and the chi-squared test or Fisher’s exact test was used for the comparison of category variables between groups, as appropriate.

The effects of physical function on the rate of cognitive change were analyzed using the linear mixed effects models, which take into account the dependence of repeated observations by participants, an important feature of longitudinal analyses. In addition, the linear mixed effects models can handle missing data due to dropouts during the follow-up period by implying missing data using random maximum likelihood methods so a researcher can use all available data during follow up studies [35, 36].

To test the changes in MMSE and DSST in the linear or non-linear model, we performed likelihood-ratio tests to examine whether models including the time and time-squared terms fit better than those including only the time term. The results indicated that, whereas the model assuming a curvilinear linear change in DSST fit better than that assuming a linear change [χ^2^(4) = 102.1, *P* < .001], MMSE changed linearly. Therefore, we only modeled non-linear change in DSST.

The models used in the present study included fixed terms for the intercept (baseline performance for an individual with a grand-mean value of all covariates), physical parameters (gait speed Q1 or handgrip strength Q1, based on the lowest quintile as reference), time (time in years since baseline), time-squared terms (only for DSST), and a physical parameter x time interaction term. Age, gender, education years, marital status, smoking status, BMI, IADL, depressive symptoms, CCI (at baseline) and their interaction terms were included as covariates. However, we did not include interactions between independent variables (i.e., physical function and covariates) and time-squared variables in modeling DSST. If we included the latter interactions, none were significant and the model fit worsened in comparison with the model that only included interactions between independent variables and time. Random effects of the intercept (baseline performance) and slope (change over time) were calculated using an unstructured covariance matrix with residual maximum likelihood. The terms of primary interest in this study were physical parameters x time interactions, which reflect whether the levels of physical performance at baseline differed in the rate of change in cognitive functions over time. For all tests, a two-tailed *p*-value of < 0.05 was considered statistically significant. All statistical analyses were performed using IBM SPSS version 23.

## Results

### Baseline characteristics

Overall, 1096 participants were enrolled from the second wave, which was used as the baseline for analyses. Table 1 compares the demographic characteristics of study participants with different gait speed based on quintiles. There were 33 participants having no record for gait speed, and in total, 1063 participants with a gait speed measurement were analyzed, as shown in Table 1. The results of the analysis showed that participants with slower gait speed were older (*P* < 0.001), had less education (*P* < 0.001), showed higher depressive symptoms (*P* = 0.030), and had a higher CCI (*P* = 0.002), a lower MMSE score (*P* = 0.025), and a lower DSST score (*P* < 0.001) (Table 1). Table 2 shows the comparison of participants with different handgrip strengths. There were 15 participants without handgrip strengths measurement, and in total, the handgrip strength records of 1081 participants were analyzed, as shown in Table 2. The analysis showed that participants with lower handgrip strength were older (*P* < 0.001), had less education (*P* < 0.001), had a lower BMI (*P* < 0.001), had higher depressive symptoms (*P* = 0.009), and had a higher CCI (*P* < 0.001), lower MMSE score (*P* < 0.001), and lower DSST score (*P* < 0.001) (Table 2).

**Table 1:** Comparison of baseline demographic data according to the quintiles of gait speed

Variable	Gait Speed *N* = 1063					*P* value
	Q1 230 (21.6%)	Q2 215 (20.2%)	Q3 227 (21.4%)	Q4 199 (18.7%)	Q5 192 (18.1%)	
Age (years)	72.7 ± 5.4	70.4 ± 5.6	69.3 ± 5.4	67.1 ± 5.2	66.3 ± 4.9	< 0.001
Gender						0.684
Male	121 (52.6%)	102 (44.4%)	122 (53.7%)	98 (49.2%)	99 (51.6%)	
Female	109 (47.4%)	113 (52.6%)	105 (46.3%)	101 (50.8%)	93 (48.4%)	
Smoking status						0.124
Non-smoking	180 (78.3%)	183 (85.5%)	190 (83.7%)	169 (84.9%)	167 (87.0%)	
Smoking	50 (21.7%)	31 (14.5%)	37 (16.3%)	30 (15.1%)	25 (13.0%)	
Education (years)	10.4 ± 2.7	10.3 ± 2.5	10.8 ± 2.7	11.2 ± 2.4	11.5 ± 2.6	< 0.001
Marital status						0.060
Single	61 (26.5%)	41 (19.2%)	44 (19.4%)	36 (18.1%)	30 (15.6%)	
Married	169 (73.5%)	174 (80.9%)	183 (80.6%)	163 (81.9%)	162 (84.4%)	
BMI (kg/m^2^)	23.0 ± 3.3	22.7 ± 2.8	22.9 ± 3.0	22.6 ± 2.9	23.0 ± 2.7	0.616
TMIG-IC	4.8 ± 0.5	4.9 ± 0.3	4.9 ± 0.3	4.9 ± 0.3	4.9 ± 0.5	0.168
Depressive symptom						0.030
Yes	34 (15.6%)	24 (11.4%)	19 (8.7%)	18 (9.4%)	12 (6.4%)	
No	184 (84.4%)	186 (88.6%)	200 (91.3%)	174 (90.6%)	176 (93.6%)	
CCI	1.8 ± 2.6	1.7 ± 2.3	1.2 ± 1.8	1.4 ± 1.8	1.2 ± 1.6	0.002
MMSE	27.8 ± 1.8	28.1 ± 1.6	28.1 ± 1.6	28.1 ± 1.6	28.3 ± 1.5	0.025
DSST	37.4 ± 10.2	41.5 ± 9.7	43.2 ± 10.5	46.2 ± 11.0	48.0 ± 11.1	< 0.001

Values are presented as the mean (SD) or counts (percentages)

Gait speed in each group:

In males: Q1 as the lowest quintile and Q5 as the highest quintile; ≦1.12; 1.13–1.25; 1.26–1.37; 1.38–1.47; and > 1.48, m/s

In females: Q1 as the lowest quintile and Q5 as the highest quintile; ≦1.13; 1.14–1.22; 1.23–1.32; 1.33–1.42; and > 1.43, m/s

*BMI* body mass index, *TMIG-IC* Tokyo Metropolitan Institute of Gerontology Index of Competence, *CCI* Charlson Comorbidity Index, *MMSE* Mini-Mental State Examination, *DSST* Digit Symbol Substitution Test

The following data were missing: smoking status, *n* = 1; TMIG-IC, *n* = 5; depressive symptoms, *n* = 36; CCI, *n* = 17; DSST, *n* = 2

**Table 2:** Comparison of baseline demographic data according to the quintiles of handgrip strength

Variable	Handgrip Strength *N* = 1081					*P* value
	Q1 219 (20.3%)	Q2 218 (20.2%)	Q3 217 (20.1%)	Q4 217 (20.1%)	Q5 210 (19.4%)	
Age (years)	72.9 ± 5.0	70.9 ± 5.5	69.3 ± 5.5	67.8 ± 5.4	65.8 ± 4.9	< 0.001
Gender						0.990
Male	112 (51.1%)	111 (50.9%)	111 (51.2%)	108 (49.8%)	110 (52.4%)	
Female	107 (48.9%)	107 (49.1%)	106 (48.8%)	109 (50.2%)	100 (47.6%)	
Smoking status						0.056
Non-smoking	176 (80.4%)	180 (82.6%)	196 (90.3%)	179 (82.9%)	174 (82.9%)	
Smoking	43 (19.6%)	38 (17.4%)	21 (9.7%)	37 (17.1%)	36 (17.1%)	
Education (years)	10.4 ± 2.6	10.3 ± 2.5	10.8 ± 2.6	11.0 ± 2.8	11.3 ± 2.6	< 0.001
Marital status						0.754
Single	49 (22.4%)	46 (21.1%)	41 (18.9%)	42 (19.4%)	37 (17.6%)	
Married	170 (77.6%)	172 (78.9%)	176 (81.1%)	175 (80.6%)	173 (82.4%)	
BMI (kg/m^2^)	22.1 ± 3.5	22.8 ± 3.1	23.0 ± 2.9	23.3 ± 2.9	23.4 ± 2.6	< 0.001
TMIG-IC	4.8 ± 0.5	4.9 ± 0.4	4.9 ± 0.4	4.9 ± 0.4	4.9 ± 0.4	0.723
Depressive symptoms						0.009
Yes	35 (16.4%)	21 (10.0%)	24 (11.7%)	19 (9.0%)	12 (5.9%)	
No	178 (83.6%)	189 (90.0%)	181 (88.3%)	193 (91.0%)	193 (94.1%)	
CCI	2.0 ± 2.4	1.6 ± 2.4	1.4 ± 2.0	1.2 ± 1.7	1.1 ± 1.7	< 0.001
MMSE	27.8 ± 1.8	27.9 ± 1.7	28.0 ± 1.6	28.4 ± 1.5	28.3 ± 1.5	< 0.001
DSST	38.4 ± 10.4	39.9 ± 10.4	43.1 ± 10.0	44.6 ± 11.8	48.3 ± 10.7	< 0.001

Handgrip strength in each group:

In males: Q1 as the lowest quintile and Q5 as the highest quintile: ≦30.70; 30.71–33.90; 33.91–37.40; 37.41–41.25; and > 41.25 kg

In females: Q1 as the lowest quintile and Q5 as the highest quintile: ≦18.50; 18.51–20.90; 20.91–23.10; 23.11–25.70; and > 25.70 kg

*BMI* body mass index, *TMIG-IC* Tokyo Metropolitan Institute of Gerontology Index of Competence, *CCI* Charlson Comorbidity Index, *MMSE* Mini-Mental State Examination, *DSST* Digit Symbol Substitution Test

The following data were missing: smoking status, *n* = 1; TMIG-IC, *n* = 5; depressive symptoms, *n* = 36; CCI, *n* = 17; DSST, *n* = 2

We also examined correlations among the key variables at baseline (i.e., gait speed, handgrip strength, MMSE, and DSST) separated by gender. The intercorrelations were summarized in Appendix 1. Weak to moderate associations were observed among each other (*r* = .06–.36), which meant that the key variables were related with each other but were relatively independent within and across domains (i.e., physical and cognitive).

### Participation in the follow up periods

The mean number of included studies from the 2^nd^ to 7^th^ waves was 4.05 ± 1.94. Approximately 85% of the participants participated in the study more than once. Appendix 2 summarizes the information on follow-up participation. In total, 1096 participants were included in the analysis from the 2^nd^ wave as the baseline. Eight hundred (80.3%) participants remained in the 3^rd^ wave, and 768 (70.1%), 663 (60.5%), 562 (51.3%) and 473 (43.2%) participants remained in the 4^th^ to 7^th^ waves, respectively. The participation rates were higher in the groups with faster gait speed and higher handgrip strength from wave 3 to wave 7. Compared with those who participated at least twice from wave 2, the participants who participated in only wave 2 (*n* = 216, 19.7%) were older (72.2 ± 5.5 years versus 68.8 ± 5.7 years, *P* < 0.001), more frequently female (55.1% versus 47.6%, *P* = 0.049), had less education (10.3 ± 2.7 years versus 10.9 ± 2.6 years, *P* = 0.005), showed higher depressive symptoms (17.6% versus 9.3%, *P* = 0.001), and had a lower MMSE (27.7 ± 1.7 versus 28.1 ± 1.6, *P* = 0.001) and a lower DSST (38.7 ± 10.7 versus 43.7 ± 11.1, *P* < 0.001).

### Physical function at baseline and cognitive decline

We first performed likelihood-ratio tests to examine whether models that included the time and time-squared terms fit better than those that only included the time term. The results indicated that the models that assumed a nonlinear change in DSST and a linear change in MMSE fit well (Details are provided in the Methods section).

Next, we conducted mixed-effect models to examine whether and how physical function was associated with cognitive function over time. The results are presented in Table 3. The time term was not significant (estimate = − 0.05 and *P* = 0.606 in the gait speed model; estimate = − 0.04 and *P* = 0.710 in the handgrip strength model), but the time-squared term was significant (estimate = − 0.05 and *P* < 0.001 in the gait speed model; estimate = − 0.05 and *P* < 0.001 in the handgrip strength model) for DSST, indicating an accelerated cognitive decline over time. The time term was significant for MMSE (estimate = − 0.16 and *P* < 0.001 in the gait speed model; estimate = − 0.17 and *P* < 0.001 in the handgrip strength model), indicating a constant cognitive decline over time.

**Table 3:** Estimated 10-year cognitive decline in each gait speed and handgrip strength quintile by linear mixed effects models

Model terms	DSST		MMSE	
	Estimate (SE)	*P*-value	Estimate (SE)	*P*-value
【Gait speed】				
Intercept	40.88 (0.62)	< 0.001	27.96 (0.10)	< 0.001
Gait speed Q1^a^	Reference			
Gait speed Q2	2.41 (0.86)	0.005	0.16 (0.14)	0.261
Gait speed Q3	2.21 (0.85)	0.010	0.14 (0.14)	0.305
Gait speed Q4	3.48 (0.91)	< 0.001	0.08 (0.15)	0.598
Gait speed Q5	3.96 (0.94)	< 0.001	0.25 (0.15)	0.097
Time^b^	−0.05 (0.09)	0.606	−0.16 (0.02)	< 0.001
Time-squared^b^	−0.05 (0.01)	< 0.001	–	
Gait speed Q1 x time	Reference			
Gait speed Q2 x time	0.14 (0.09)	0.140	0.02 (0.03)	0.421
Gait speed Q3 x time	0.15 (0.09)	0.101	0.05 (0.03)	0.062
Gait speed Q4 x time	0.13 (0.09)	0.146	0.02 (0.03)	0.590
Gait speed Q5 x time	0.28 (0.09)	0.003	0.05 (0.03)	0.078
【Handgrip Strength】				
Intercept	41.57 (0.63)	< 0.001	27.93 (0.10)	< 0.001
Handgrip strength Q1^c^	Reference			
Handgrip strength Q2	0.49 (0.86)	0.567	0.07 (0.14)	0.614
Handgrip strength Q3	1.64 (0.88)	0.063	0.07 (0.14)	0.628
Handgrip strength Q4	2.15 (0.90)	0.017	0.38 (0.14)	0.008
Handgrip strength Q5	3.42 (0.95)	< 0.001	0.23 (0.15)	0.127
Time^b^	−0.04 (0.09)	0.710	−0.17 (0.02)	< 0.001
Time-squared^b^	− 0.05 (0.01)	< 0.001	–	
Handgrip strength Q1 x time	Reference			
Handgrip strength Q2 x time	0.07 (0.10)	0.439	0.04 (0.03)	0.189
Handgrip strength Q3 x time	0.11 (0.09)	0.259	0.05 (0.03)	0.060
Handgrip strength Q4 x time	0.20 (0.09)	0.033	0.05 (0.03)	0.082
Handgrip strength Q5 x time	0.20 (0.10)	0.040	0.06 (0.03)	0.039

All models were adjusted by covariates (age, gender, education years, marital status, smoking status, body mass index, Tokyo Metropolitan Institute of Gerontology Index of Competence, depressive symptoms and Charlson Comorbidity Index) and their interactions with time. The covariates were grand-mean centered. However, these estimates were not presented, and only the estimates of interest (intercept, time, and their interactions with physical function) are shown

In males: Q1 as the lowest quintile and Q5 as the highest quintile; ≦1.12; 1.13–1.25; 1.26–1.37; 1.38–1.47; and > 1.48, m/s

In females: Q1 as the lowest quintile and Q5 as the highest quintile; ≦1.13; 1.14–1.22; 1.23–1.32; 1.33–1.42; and > 1.43, m/s

In males: Q1 as the lowest quintile and Q5 as the highest quintile: ≦30.70; 30.71–33.90; 33.91–37.40; 37.41–41.25; and > 41.25 kg

In females: Q1 as the lowest quintile and Q5 as the highest quintile: ≦18.50; 18.51–20.90; 20.91–23.10; 23.11–25.70; and > 25.70 kg

*SE* standard error, *MMSE* Mini-Mental State Examination, *DSST* Digit Symbol Substitution Test

Among a sample of 1096 adults, those with missing values in physical and cognitive functions and covariates at baseline were excluded from the analyses. Thus, the sample sizes varied according to the models: in the gait speed models, *N* = 1006 for DSST, and *N* = 1007 for MMSE; in the grip strength models, *N* = 1022 for DSST, and *N* = 1024 for MMSE

^a^Gait speed in each group

^b^Years since baseline. Time-squared was included in modeling non-linear change of DSST

^c^Handgrip strength in each group

We will continue to report significant associations between the intercept or linear slope with physical function to indicate whether and which physical function indicators buffered cognitive decline. In the gait speed model, the group in the lowest quintile (Q1) showed significantly lower DSST scores than the other groups (estimate = 2.41 and *P* = 0.005 for Q2; estimate = 2.21 and *P* = 0.010 for Q3; estimate = 3.48 and *P* < 0.001 for Q4; estimate = 3.96 and *P* < 0.001 for Q5), but the differences in the MMSE scores were absent. The term for the gait speed × time interaction in Q5 was significant for DSST decline (estimate = 0.28 and *P* = 0.003), showing that the subjects in the lowest quintile group of gait speed had a greater DSST decline over time than those in the highest quintile group.

**Fig. 1:**
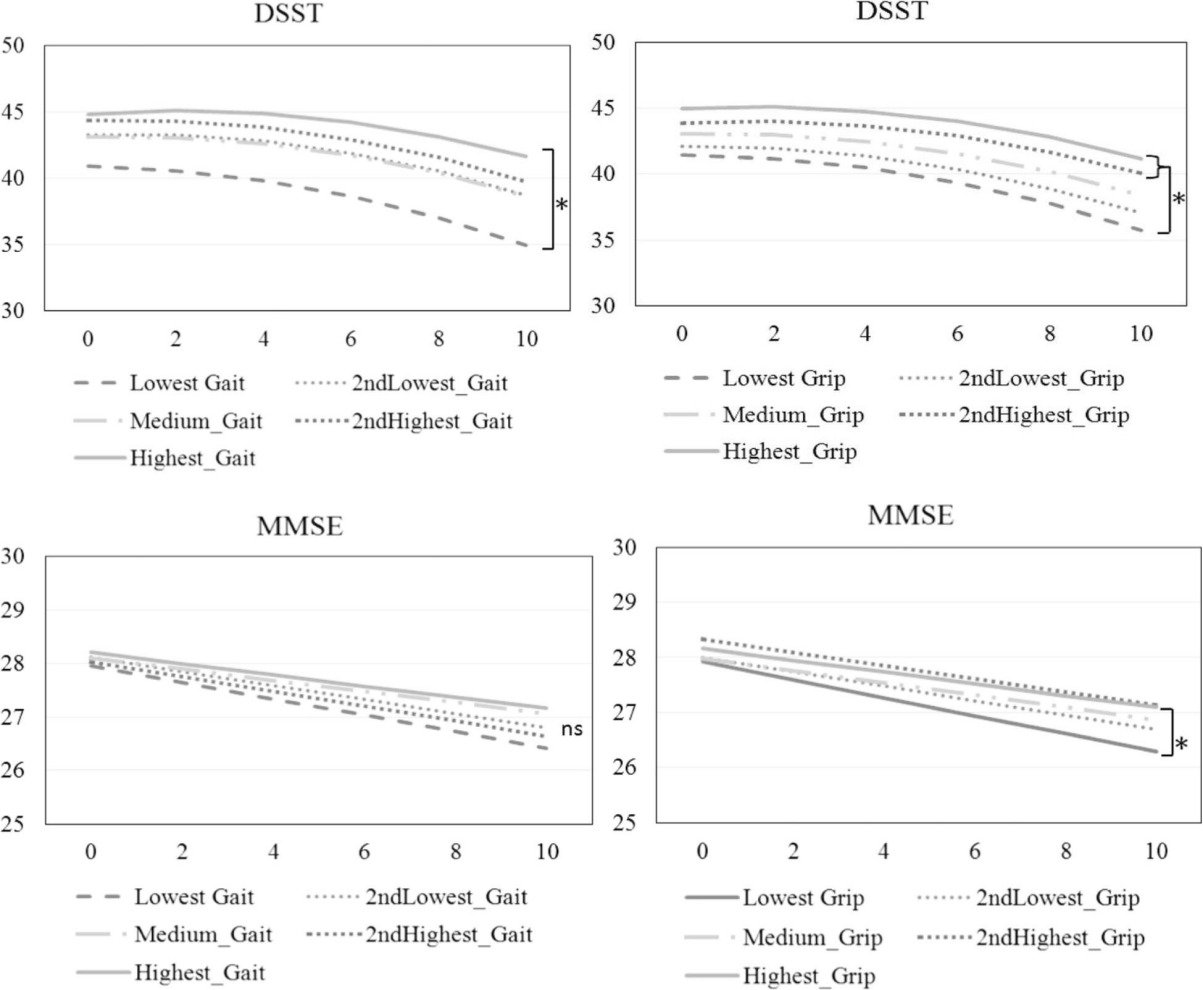
Model-predicted 10-year cognitive decline using the MMSE and DSST in the different gait speed and lowest handgrip strength quintile groups. ns: no significant. *: *P*-value < 0.05

In the handgrip strength model, those with the lowest handgrip strength (Q1) showed significantly lower DSST scores than those in Q4 and Q5 (estimate = 2.15 and *P* = 0.017 for Q4; estimate = 3.42 and *P* < 0.001 for Q5), but a significant difference was only observed for Q4 in the MMSE score (estimate = 0.38 and *P* = 0.008). The terms for the handgrip strength × time interaction in Q4 and Q5 were significant for DSST (estimate =0.20 and *P* = 0.033 for Q4; estimate = 0.20 and *P* = 0.040 for Q5). For MMSE, significance was found in Q5 (estimate = 0.06 and *P* = 0.039). The differences in cognitive decline between high and low physical function groups were shown in Fig. 1.

Regarding DSST, we failed to observe any significant independent variables (i.e., physical function and covariates) associated with the time-squared term and did not include the interactions with the time-squared term (results not shown).

## Discussion

To the best of our knowledge, the present longitudinal study is the first to examine differential associations of gait speed and handgrip strength with the DSST and MMSE as indicators of long-term cognitive decline among community-dwelling older people in Japan. In addition, in the present study, the participants were invited into the study based on a random sample stratified by age and gender and the follow up period was up to 10 years. Most other longitudinal studies show that physical function can predict cognitive decline by using global cognitive tests, such as MMSE, but only a few have explored executive functional decline by using the DSST at the same time in their studies of community populations [10, 11, 13]. The DSST is a specific measure of executive cognitive function, working memory, processing speed, and visuospatial attention and might be a more sensitive measure of slight cognitive changes than the MMSE when seeking to detect early cognitive decline among community-dwelling older people [18]. Moreover, the current consensus for gait speed and handgrip strength as key determinants of physical frailty and sarcopenia suggested the use of the quintile or quartile as the cut-off points, and our present study used the lowest quintile of physical function compared with the others to explore the prediction of the 10-year cognitive decline [25, 26]. Our study demonstrated that at baseline, a slow gait speed and low handgrip strength were significantly associated with lower DSST scores, but not MMSE scores except between Q1 and Q4 for handgrip strength. Moreover, the results revealed both gait speed and handgrip strength were associated with changes in cognitive function. When comparing participants with the slowest gait speed, those with the fastest gait speed (Q5) exhibited a less steep cognitive decline as measured by DSST. On the other hand, when comparing participants with the weakest grip strength, those with the strongest handgrip strength (Q5) showed a less severe cognitive decline as measured by MMSE in addition to DSST.

Our study showed that poor physical function was associated with cognitive decline, which was compatible with previous studies (15–17). When aging, health behavior and age-related biological factors generally underlie normative age-related decline in physical function, which in turn could lead to cognitive decline. Current physical activity guidelines also reported that to do multicomponent physical activity including balance training and aerobic and muscle-strengthening activities could improve cognition and reduce the risk of dementia [37]. Furthermore, poor physical condition is associated with depressive symptoms and limited social interactions and leisure activities [38–40]. Subjects with depressive symptoms were reported have higher risk for developing cognitive impairment than those without [41]. Moreover, some shared pathogenic factors have been found between low muscle strength and impaired cognitive function. High oxidative stress, high inflammatory markers, insulin resistance and low sex steroid levels were reported to contribute to both muscle loss and cognitive decline and might account for the association between poor physical function and cognitive decline [42–44].

Our result supports previous findings that gait speed is correlated with the DSST scores decline [45–47]. Best et al reported that a slow walking speed was more strongly correlated with cognitive decline in DSST rather than in global cognition [45]. Walking and gait may rely on motor and cognitive functions, which may share similar neural and pathological mechanisms [15]. The networks in the brain that control walking involve regions that are responsible for attention, executive and visuospatial functions, as well as regions that control motor tasks. Previous research has demonstrated that the cerebellum, basal ganglia, hippocampus, and parietal and frontal cortices are related to gait and executive functions [48–50]. To unravel the question of whether declines in gait speed and executive function are closely associated with aging, the time-varying nature of physical function should be further taken into consideration.

Our study found that low handgrip strength was associated with the cognitive decline with MMSE as well as DSST [12, 13, 51–53]. Low handgrip strength and executive functional decline might also share a similar *etiopathogenesis*. Skilled hand movements and handgrip strength are associated with the primary motor area, supplementary motor area, dorsal and ventral parts of the premotor cortex area and cerebellum, as shown in neuroimaging studies, and these may also play a role in higher cognitive functions [54–56]. The correlation between poor physical condition and global cognitive decline might also be explained by reduced gray and white matter volumes in multiple brain regions and white matter hyperintensities and greater activation in pre-supplementary motor, pre-dorsal motor, rostral cingulate and prefrontal cortex by a functional MRI study [57, 58]. However, in our study, we only found that low handgrip strength was correlated with global cognitive decline, but not slow gait speed. Alfaro-Acha et al., also reported that handgrip strength may be an early marker of subsequent cognitive decline [51]. We further performed a follow-up analysis to examine whether gait speed and handgrip strength had independent effects on changes in DSST. After including both physical function parameters simultaneously, the model revealed that only gait speed was associated with the decline in DSST (estimate = 0.25, *P* = 0.008 for Q5 in reference to Q1). The results of the follow-up analysis would indicate a unique linkage between gait speed and DSST. A recent review (Tian et al., 2017) also suggests that gait can be associated with specific brain areas important for sensorimotor function. However, these underlying mechanisms have not been unraveled yet.

Despite all the efforts that went into designing and carrying out this study, there were still several limitations. First, none the participants were diagnosed with mild cognitive impairment and dementia, and there were 37 participants with cognitive impairment (according to the definition of MMSE< 24) at baseline who were excluded for analysis. Therefore, the results are not relevant among those with mild cognitive impairment and dementia. However, all the participants were recruited by matching age and gender in the community, and therefore the results of the present study could be applied to the community-dwelling older adults without cognitive impairment. Second, the statistical models in the present study did not include potential covariates that might influence cognitive decline, such as visual and auditory functions; however, we adjusted several important factors as much as possible to minimize this confounding bias. Third, 19.7% of the participants who participated in the baseline survey or wave 2 only had more impaired physical and cognitive functions. We assumed that this attrition meant responses were missing at random. However, participants with impaired cognitive function also tended to drop out from the follow up surveys. Therefore, we might underestimate cognitive decline when using a cognitively intact sample. Although the results indicated a non-linear change in DSST, we found no significant associations with the time-squared term. Future research needs to explore potential protective factors that buffer accelerated cognitive decline.

## Conclusions

A slow gait speed could predict 10-year cognitive decline using DSST, and a low handgrip strength could predict 10-year cognitive decline using MMSE in addition to DSST. Thus both physical measures are lined to cognitive decline but there may be different mechanism between brain and physical functions.

## Data Availability

The datasets generated and analyses performed during the current study are not publicly available due to the consent requirement of participants, but sex and age decade-stratified descriptive data are available from the corresponding author on reasonable request.

## Appendix 1

**Table 4:** Intercorrelations among the key variables at baseline separated by gender

	1	2	3	4
1. Gait speed	–	.37*	.06	.31*
2. Handgrip strength	.33*	–	.15*	.34*
3. MMSE	.06	.15*	–	.36*
4. DSST	.31*	.34*	.36*	–

The results for male are below and those for females are above the diagonals

*MMSE* Mini-Mental State Examination, *DSST* Digit Symbol Substitution Test

**P* < .001

## Appendix 2

**Table 5:** Comparison for the characteristics of those participating only once in 2^nd^ wave and others

	Maintain in 3rd wave *n* = 880 (80.3%)	Only participation in 2nd wave *n* = 216 (19.7%)	*P* value
Age (years)	68.8 ± 5.7	72.2 ± 5.5	< 0.001
Gender			0.049
Male	461 (52.4%)	97 (44.9%)	
Female	419 (47.6%)	119 (55.1%)	
Smoking status			0.712
No smoking	735 (83.6%)	182 (84.7%)	
Smoking	144 (16.4%)	33 (15.3%)	
Education	10.9 ± 2.6	10.3 ± 2.7	0.005
Marital status			
Single	174 (19.8%)	48 (22.2%)	.422
Married	706 (80.2%)	168 (77.8%)	
BMI (kg/m^2^)	22.8 ± 2.9	23.2 ± 3.6	0.110
TMIG-IC	4.9 ± 0.4	4.8 ± 0.6	0.211
Depressive symptoms			0.001
Yes	79 (9.3%)	36 (17.6%)	
No	774 (90.7%)	169 (82.4%)	
CCI			
MMSE	28.1 ± 1.6	27.7 ± 1.7	0.001
DSST	43.7 ± 11.1	38.7 ± 10.7	< 0.001

*BMI* body mass index, *TMIG-IC* Tokyo Metropolitan Institute of Gerontology Index of Competence, *CCI* Charlson Comorbidity Index, *MMSE* Mini-Mental State Examination, *DSST* Digit Symbol Substitution Test

## Abbreviations

BMI: Body mass index
CCI: Charlson Comorbidity Index
CESD: Center for Epidemiologic Studies Depression Scale
DSST: Digit Symbol Substitution Test
IADL: Instrumental activities of daily life
JWAIS-R-SF: The Japanese Wechsler Adult Intelligence Scale–Revised Short Form
MMSE: Mini-Mental State Examination
NILS-LSA: The National Institute for Longevity Science – Longitudinal Study of Aging
SD: Standard deviation
TMIG-IC: The Instrumental Self-Maintenance subscale of the Tokyo Metropolitan Institute of Gerontology Index of Competence
